# Integrated Band-Stop Filter-Based 1.8 GHz RF Detection System for Sensitivity and Efficiency Enhancement in IoT Energy Harvesting

**DOI:** 10.3390/mi17060701

**Published:** 2026-06-08

**Authors:** Naimul Hasan, Kousik Roy, Subhadip Das, Parthapratim Sarkar

**Affiliations:** 1 Department of Electronics & Communication Engineering, Sanaka Educational Trust’s Group of Institutions, Durgapur 713212, West Bengal, India; naimulhasan1984@gmail.com; 2 Department of Computer Science & Engineering, Bengal College of Engineering and Technology, Durgapur 713212, West Bengal, India; sd.kgec.mtech@gmail.com; 3 Department of Engineering and Technological Studies, Kalyani University, Kalyani 741235, West Bengal, India; ppsarkar@klyuniv.ac.in

**Keywords:** energy harvesting, IoT, RF detector, sensitivity, efficiency, band stop filter

## Abstract

The growing expansion of the Internet of Things and wireless sensor networks has created an urgent demand for compact and reliable radio frequency energy-harvesting circuits. This study introduces the design, simulation and extensive performance of a high-efficiency single band radio frequency detection system optimized for 1.8 GHz operation. The detector is realized on a Rogers RO4003C substrate and employs the SMS7630-079LF Schottky diode, selected for its excellent detection capability and economic viability. The introduction of this filtering stage effectively suppresses undesired harmonic components produced during the rectification process, thereby improving the sensitivity and overall power conversion efficiency of the system. The circuit shows a sensitivity of 1.8 mV for every dBm through its simulation tests. The system shows increased sensitivity to 2.2 mV/dBm because of the band stop filter implementation. The system reaches its peak power conversion efficiency of 65.28% at a 1.5 kΩ load, which makes it suitable for applications that require low-power energy harvesting. These combined attributes establish the developed 1.8 GHz detector as a strong candidate for next-generation energy harvesting modules, self-powered sensor networks and intelligent embedded computing platforms within the expanding domain of the Internet of Things.

## 1. Introduction

The ongoing developments in wireless communication technology and the rapid increase in the Internet of Things have led to the transformation of modern electronics into a connected and smart ecosystem. At present, billions of devices are capable of communicating and interacting with one another instantly, thereby creating a large network of sensing, computing and decision-making units that are located in homes, industries, cities and healthcare systems. The connected devices need to operate all the time in a reliable manner with very little human intervention. Unfortunately, a majority of these devices still rely on traditional electrochemical batteries which have limited energy density, short life span, and pose a significant threat to the environment. The situation gets worse when batteries have to be replaced or recharged periodically, especially if the devices are in remote, hard-to-reach or unsafe locations. Thus, the constraints associated with the use of batteries have promoted the search for alternative power sources that would guarantee the steady operation of low-power embedded systems without the need for human intervention [1]. The method of ambient energy harvesting has become the most trustworthy and green solution to limit the power supply issues of low-energy circuits. It utilizes the tiny amounts of energy that are naturally present in the environment and turns them into usable electrical power for low-energy circuits. Among the different sources of ambient energy, the sun, mechanical vibrations, heat difference and radio frequency electromagnetic waves are the main ones. Radio frequency energy harvesting is one that has gained more and more interest over the past years owing to its continuous availability, accessibility throughout the world and independence from environmental factors like sunlight or motion. Radio frequency signals are naturally emitted by many sources like mobile base stations, wireless routers, TV broadcasting towers and Bluetooth communication devices. The technique of turning this widely available electromagnetic energy into power opens up the possibility of making self-sustainable sensor nodes and smart devices for application areas like wireless sensor networks for environmental and structural monitoring, precision agriculture and biomedical instrumentation [2]. A typical RF energy harvesting architecture usually has four main parts. The first part is the antenna that picks up the electromagnetic waves from the environment. The second part is the impedance-matching network that guarantees the maximum power transfer between the antenna and the rectifying circuit. The third part, named the rectifier or RF detector, transforms the alternating radio signal into a direct current voltage that can either charge a storage unit or directly power the electronic load. The last part is the energy storage or regulation section that stabilizes and conditions the output voltage. Among the various components that make up the blocks, the rectifier is of utmost importance because it has a considerable influence on the whole system’s efficiency, sensitivity and reliability. Thus, the rectifier circuit design has become an important aspect of energy harvesting research [3]. The working of a rectifier circuit is really dependent on some factors like diode choice, matching network design, harmonic suppression, the nature of the substrate used and layout. At low power levels typical of ambient radio, conventional rectifiers are the ones that show limited efficiency and non-linearity. To overcome these problems, current works are focusing on adding filtering elements and impedance-controlled configurations directly into the rectifier circuit so that conversion efficiency and output stability are both improved. The merging of the resonant filters is able to reduce the rectification-process-generated harmonic frequencies and thereby enhance signal clarity and input level softness. The whole process and advances mentioned above are extremely necessary for the implementation of self-powered computing components and communication systems with smart features that are expected to be in the IoT operating framework. The frequency of 1.8 gigahertz is particularly important in this case because it falls within a range that is very much in demand for cellular communication and also provides a steady and adequate amount of energy for harvesting. The designing of effective detectors in this frequency range allows power to be drawn from the existing communication network and without the use of any additional energy sources. The allocation of this band for power also makes it easier to combine with the latest embedded and IoT devices that already work within or close to this frequency. Therefore, creating a high-efficiency single-band radio frequency detector operating at 1.8 GHz is a sensible and progressive step toward ensuring the long-term viability of energy-autonomous electronic systems.

The system receives incident RF signals through an impedance-matched network which connects to the rectifying circuit. The system uses a low-pass RC filter to smooth the rectified signal which produces a usable DC output. The system functions as a RF detection and rectification circuit but it needs an external antenna or RF source to operate. Our research work mainly concentrates on developing a high-sensitivity RF detector and a rectifying unit. The device achieves efficient rectification of low-power RF signals through its input port by using the square-law operating mode of the Schottky diode. The multi-stop-band band stop filter implementation enables the system to block higher-order harmonics while improving RF voltage usage at the diode which leads to better RF-to-DC conversion efficiency. The study assumes that RF input enters through a matched transmission line which allows it to evaluate rectifier design and performance. The design and performance evaluation of a high-efficiency single-band radio frequency detector operating at 1.8 GHz for energy harvesting and embedded computer applications are the main objectives of the current study. A Rogers RO4003C substrate chosen for its high stability at microwave frequencies and low dielectric loss has been used to create the circuit. Because of its quick switching speed, tiny junction capacitance and low threshold voltage, an SMS7630 LF Schottky diode has been used to ensure effective conversion even with weak input conditions. There have been two design configurations examined. The first has a traditional detector topology but the second incorporates a band stop filter based on a stepped-impedance resonator into the rectification path. The circuit’s filter’s integration facilitates the complete removal of the harmonic components that were produced during the rectification process. The benefit of the increase in linearity, sensitivity and the overall efficiency of power conversion is obtained directly. Various load conditions have been simulated in the Advanced Design System environment for performance evaluation of the detector. The filtering element-free configuration is sensitive to 8.69 mV while the filtered one has 10.38 mV sensitivity, thus making it evident that harmonic suppression has a huge impact on sensitivity. The optimized circuit produced a dc output voltage of 0.95 V at 0 dBm input power with 9.53 kΩ load resistance and 65.28% maximum power conversion efficiency at 1.5 kΩ load. This clearly indicates that the filtering network has a major impact on circuit performance but does not add to its size or its complexity. Comparing the new single-band detectors on the basis of sensitivity and cost shows that the new design is a compact, energy-efficient, and functionally reliable alternative for self-powered sensor nodes and low-energy embedded computing platforms.

This paper presents an innovative solution that combines a compact multi-stop-band band stop filter which uses stepped impedance resonators and an open-stub structure with a single Schottky diode detector. This system enables fundamental and higher-order harmonics suppression up to the fourth-order harmonic, which improves sensitivity and linearity while keeping the system design simple and compact and cost-effective.

This research’s main contributions and highlights are as follows:

- Creation of a single-band radio frequency detector that works well at 1.8 GHz and is perfect for Internet of Things and embedded energy-harvesting applications.
- Adding a stepped impedance resonator-based band stop filter to the rectifying section to block unwanted harmonic frequencies, which improves linearity and sensitivity.
- Use of a low-loss Rogers RO4003C substrate and an SMS7630 LF Schottky diode to make sure that detection is very efficient and cheap.
- Showing that the power conversion efficiency and sensitivity have improved with 65.28% efficiency and 10.38 millivolt sensitivity at 0 dBm input power.
- A comparison with standard detector designs that shows better harmonic rejection, better impedance matching and higher energy conversion without making the circuit more complicated.
- The system operates effectively with self-powered Internet of Things nodes and embedded computing systems which can function without batteries in power-efficient mode.

This research advances energy-autonomous electronic systems by demonstrating that harmonic suppression and optimization improve sensitivity and energy conversion efficiency in compact detectors. The proposed structure impedance is a good way to power small embedded computing modules, wireless telemetry systems and smart sensing devices in modern Internet of Things ecosystems. The addition of this detector to future energy harvesting networks will enable distributed wireless systems to operate continuously without maintenance while decreasing their dependence on conventional power systems. The proposed detector is a good candidate for future wireless energy harvesting systems because it has a small size, better harmonic suppression and a high conversion efficiency. The system establishes a foundation which enables permanent embedded electronics and smart sensor networks to achieve reliable energy independence throughout connected environments.

### 1.1. Role of Schottky Diodes in RF Detection

Various rectifying devices have been employed in RF-to-DC conversion such as MOSFETs, tunnel diodes and Schottky barrier diodes. Schottky diodes stand out as the most favored option due to their low forward voltage drop, rapid switching speed and minimal reverse recovery time, rendering them ideal for high-frequency and low-power applications. These characteristics are especially important for weak input power levels where small signal amplitude must be efficiently converted to DC voltage [4]. The SMS7630-079LF (Skyworks Solutions, Inc., Irvine, CA, USA), HSMS285x (Avago Technologies (Broadcom Inc.), San Jose, CA, USA) and SMS7621006LF (Skyworks Solutions, Inc., Irvine, CA, USA), Schottky diodes which are available in commercial markets are commonly used in RF rectifiers and energy-harvesting circuits because they possess low junction capacitance combined with high detection sensitivity. The practical implementations face obstacles which prevent them from achieving their desired efficiency levels. The diode’s nonlinear current–voltage characteristic produces higher order harmonics, which cause rectification to generate electrical noise that reduces DC output quality while disrupting impedance matching between the antenna and the rectifier. This mismatch causes reflection losses which result in decreased usable output voltage during low input power conditions. The unwanted harmonics can also radiate back through the antenna, causing interference with nearby communication bands and lowering the overall system performance. Consequently, optimizing diode selection, impedance matching and harmonic suppression is crucial for attaining high sensitivity and power conversion efficiency [5].

### 1.2. Challenges in Existing Rectifier Designs

Several rectifier topologies have been proposed to improve conversion efficiency and output voltage. Single-series rectifiers, voltage doublers and multi-stage voltage multipliers are among the most popular designs. Every structure has its benefits but it also has drawbacks. Although it is small and easy to construct, the single-series diode rectifier has poor harmonic rejection and low output voltage. Due to additional losses, complex matching networks and parasitic capacitance from several diodes, voltage doublers and multi-stage rectifiers raise the output voltage but reduce efficiency [6]. The lack of harmonic suppression networks is a significant disadvantage of conventional rectifier circuits. Harmonic frequencies are created by the diode’s nonlinear nature and spread throughout the circuit. Energy loss and low sensitivity may arise from these harmonics, radiating through the antenna once again if they are not filtered. In addition to decreasing linearity, strong harmonic content disrupts the detector’s reaction to weak signal levels. Therefore, in order to retain correct response and increase linearity without increasing circuit size or complexity, harmonic control methods must be included [5]. Frequency-selective structures have been investigated recently as a potential solution to these problems. Band stop filters, impedance matching networks and harmonic traps have all been included into rectifier designs in a number of studies. Although these techniques enhance performance, many of them need multilayer substrates or massive filter structures which raise manufacturing costs and restrict compactness [4]. Therefore, next-generation IoT devices need an efficient harmonic suppression method that is small, effective, and simple to construct.

### 1.3. Motivation for Band Stop Filter (BSF) Integration

A single-band RF detector integrated with a stepped impedance resonator-based band stop filter is introduced by this work as a means of overcoming the limitations of the existing rectifiers. A band stop filter is a circuit that selects frequencies based on the main operating frequency it passes and the specific harmonic frequencies it rejects. The system achieves better circuit efficiency through its capacity to reduce harmonic interference and improve matching. The stepped impedance resonator brings in a compact and versatile approach in filter design for the band stop [7]. The stop band can be precisely controlled through the tuning of the impedance section ratios and length of both the high- and low-impedance components. The SIR-based BSF used in this work efficiently diminishes the second and third harmonics while occupying a small area that is compatible with planar layouts. The integration of this BSF with the detector circuit leads to a reduction in distortion, improvement in linearity and increase in power conversion efficiency [8]. The paper examines two configurations for the detector which include a system without BSF and another system that uses SIR-based BSF. The simulation results show that BSF integration provides multiple benefits. The detector without BSF registers a sensitivity of 1.8 mV/dBm while the detector with BSF integrated shows a much higher sensitivity of 2.2 mV/dBm. The significant performance increase shows how harmonic suppression works to improve results for low-power RF signals.

### 1.4. Design Considerations and Substrate Selection

The 1.8 GHz frequency is selected for the operation of the detector which is a typical of GSM communication and IoT gateway systems. The circuit design indicates the use of microstrip transmission lines for matching impedances and routing signals because these transmission lines are simple to use and manufacture and work well with circuit board technology. Microstrip circuits allow smooth integration of filters and matching networks on a single substrate, which results in a compact and low-cost design suitable for large-scale production. The substrate used for fabrication has a strong influence on circuit performance at high frequencies. The dielectric constant, thickness and loss tangent are the main parameters that influence the impedance, signal attenuation and radiation. In this paper, Rogers RO4003C was chosen as the substrate due to its stable dielectric constant of 3.55, a low loss tangent of 0.0027 and a moderate thickness of 0.817 mm. These characteristics guarantee low dielectric loss, high impedance accuracy and consistent performance at microwave frequencies. In addition, the substrate has good manufacturability and low cost which makes it an ideal choice for the mass production of IoT-powered energy harvesting systems.

### 1.5. Overview of the Proposed Work

The comprehensive design, modeling and performance analysis of two 1.8 GHz single-band RF detectors are presented in this paper. The second detector has a band stop filter based on a stepped impedance resonator to reduce harmonics, whereas the first detector is a normal detector without any filter. The Advanced Design System software (v2019) is used to simulate the circuits and analyze their performance in terms of power conversion efficiency, sensitivity, and return loss. The suggested detector has a load resistance of 9.53 kΩ and an output DC voltage of 0.95 V at 0 dBm input power. At a load of 1.5 kΩ, a maximum power conversion efficiency of 65.28% was achieved. In terms of sensitivity, impedance matching and harmonic suppression, the detector with BSF performs better than the one without. The improvement in sensitivity from 1.8mV/dBm to 2.2 mV/dBm demonstrates the effectiveness of the BSF in enhancing detector response under low input power conditions. The proposed design also compares favorably with existing single-band detectors by offering high efficiency, compact size and low cost, which make it suitable for self-powered IoT and embedded systems.

## 2. Related Work

An RF-to-DC conversion efficiency of around 20% (with an output voltage of 97 mV) was measured by the authors in [9] at an incoming power level of −20 dBm and a load resistance of 4.7 kΩ. The best conversion efficiency of 52% was recorded in [10] for a rectenna operating at 2.45 GHz with an input power of 0 dBm. In order to achieve a maximum efficiency of 55.6% and an output DC voltage of 1.19 V under 0 dBm input (simulation only), [11] describes a rectifier design that uses an impedance-matching network made up of a 60° radial stub in series with a single stub. The rectenna was suggested for low-power electronic applications in [12], where it once again reached a conversion efficiency of up to 52% at 2.45 GHz and input power of 0 dBm. An RF energy harvester working at 5.8 GHz for wireless sensor nodes is the main topic of [13]. The rectifier’s theoretical design process is presented, and the simulation results show a strong theoretical correspondence. While storing energy in a capacitor, the power-management circuit in that work increases and controls the DC output. The stated efficiencies re 24% at −10 dBm and 47% at −5 dBm input. A battery-free railway monitoring system based on RF energy harvesting with a three-stage Dickson voltage multiplier is proposed in [14]. The device achieves a maximum energy-conversion efficiency of 25% and 500 working cycles per second when triggered by a specialized radio frequency power source at a maximum distance of 2.3 m. To achieve outstanding RF-to-DC transformation efficiency at low input power, [15] proposes an L-section matching network in conjunction with a voltage-doubler circuit. With 52% efficiency at 0 dBm input, the results exhibit return-loss and realized gain characteristics at the ISM band. Reference [16] describes a wearable rectenna intended for flexible Internet of Things applications that operates at 2.45 GHz with a maximum conversion efficiency of 55.7%; it is built on a graphene-film substrate. In order to improve rectification efficiency under low incident power, the authors of [17] choose the SMS7630 diode and place it on a rectifier. The total rectification performance of a rectifier operating in the 5.8 GHz ISM band and a six-element patch receiving array with inter-element spacing smaller than half wavelength is simulated and evaluated. A high-efficiency rectifying circuit using multi-section impedance conversion and harmonic suppression is shown in [18]. In order to reduce fundamental and higher-order harmonics, the design incorporates a matching filter network at the DC output and employs a shorted stub to compensate for the diode’s capacity. At 5.8 GHz, the efficiency is stated to be 59.6%. A rectifier circuit is examined in [19], spanning the input power range of −20 dBm to +20 dBm, and a maximum measured efficiency of 53.34% is attained at 7.4 GHz with a load resistance of 1 kΩ. Reference [20] explores low-power RF energy harvesting systems based on Schottky barrier diodes and looks at the connection between circuit temperature and RF-to-DC power conversion efficiency, which is directly related to the non-linear behavior of the metal–semiconductor junction. A dual-voltage rectifier architecture with a specially designed matching network is suggested in [21] to maximize the efficiency of RF-to-DC conversion. Because of its high non-linearity and temperature-insensitivity characteristics, an asymmetric spacer-layer tunnel diode (ASPAT) is used as the active rectifier in [22]. A novel low-power class E F2 shunt rectifier and voltage-doubler that uses a RO4003C substrate is shown in [23]. With a constant DC voltage of 3.2 V at Pin = 0 dBm and RL = 8 kΩ, the suggested voltage-doubler has an experimental peak efficiency of 57% while working in two bands of 650 MHz and 900 MHz. In [24], a rectenna harvests 4 mJ in 32 s from an input density of 16.6 µW/cm^2^ with an efficiency of 4.8%, including in-body losses and transient shadowing. A Wi-Fi band energy-harvesting rectifier that maximizes energy conversion efficiency while preserving portability and affordability is shown in [25]. Using a semi-lumped matching network on an inexpensive FR4 substrate, the device achieves 55% efficiency at +8 dBm and 47% efficiency at 0 dBm in 263 mm^2^. In [26], a rectifying circuit with a voltage-doubler architecture built for 5.8 GHz achieves 41% efficiency at 0 dBm input. The simulation findings in [27] indicate that when the ideal load resistance is 501 Ω, the highest RF-to-DC conversion efficiency is almost 58% at an RF power density of 0.233 W/m^2^. Lastly, the findings in [28] demonstrate that RF-DC power rectification efficiency sustains about 49.5% to 55% in the input power range of −20 dBm to −10 dBm, with indoor and outdoor output DC voltages of 429.8 mV and 463.1 mV, respectively. For ambient energy-harvesting applications that need a greater supply voltage in ambient surroundings, mechanism and matching strategies are suggested. In [29], a rectenna-based RF energy-harvesting system that transforms ambient electromagnetic waves into DC current is described, along with a number of ambient energy recovery sources. At 0 dBm input power at 2.45 GHz, the rectifier on ADS achieves 54% efficiency, generating 732 mV of DC voltage. According to the research, voltage-doubler topologies are better for small designs without matching filters, and two matched voltage doublers perform similarly in terms of conversion efficiency and DC voltage. A variety of frequencies, topologies, substrates, and diode technologies have been studied in the past, with efficiency usually falling between 20% and 60% under moderate to strong input circumstances. However, it is still difficult to achieve great efficiency at extremely low input power levels in low-cost, small configurations. Recent studies have reported RF detectors and rectifiers which achieve either high sensitivity for ultralow input power detection or high conversion efficiency for moderate power operation. The research presented in [30] demonstrates improved low power detection capabilities together with enhanced energy harvesting performance, which achieves sensitivities below sub-µW input power detection threshold. The findings in [31] describe stable rectification behavior together with signal detection performance across a specified dynamic range, although system sensitivity depends heavily on the specific power operating range.

The existing evaluation process for RF microwave detector designs has concentrated on assessing RF to DC conversion efficiency while overlooking the critical sensitivity factor, which determines the ability to detect low-power RF signals. The study provides a systematic evaluation of both efficiency and sensitivity. The proposed detector achieves 65.28% RF to DC conversion efficiency at 0 dBm and a sensitivity of 2.2 mV per dBm with BSF and 1.8 mV per dBm without BSF at −35 dBm input power. The results show that the detector achieves higher efficiency together with better sensitivity and maintains stable performance throughout its complete range of input power, which makes it appropriate for use in wireless sensing and Internet of Things applications.

## 3. Rectifier Topology

### 3.1. Design of the Proposed Single-Band Detector Without Band Stop Filter

**Figure 1:**
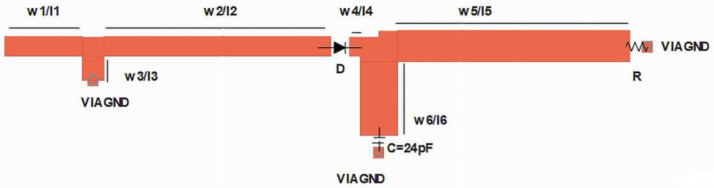
Layout of the proposed single-band detector designed at 1.8 GHz without a band stop filter.

The detector topology serves as the core of any RF energy harvesting (RFEH) system, directly influencing the RF-to-DC conversion efficiency and overall circuit performance. In the proposed design, a single-band detector operating at 1.8 GHz is developed without employing any harmonic suppression network. The schematic layout of the proposed detector is illustrated in Figure 1. The detector makes use of the SMS7630-079LF Schottky diode from Skyworks (Skyworks Solutions, Inc., Irvine, CA, USA), which was selected for its superior RF properties including quick switching speed, low forward voltage drops (≤150 mV) and good sensitivity to low input power levels. It is especially well suited for low-power rectifying circuits used in ambient energy collection because of these qualities. The diode converts the received RF signal into DC power, while a bypass capacitor (C_1_ = 24 pF, Murata (Murata Manufacturing Co. Ltd, Kyoto, Japan) (Part No. CGQ1555C1H2R4BB01) acts as a low-pass filter to smoothen the rectified DC output by attenuating high-frequency ripples. The detector network comprises six microstrip transmission lines (TL_1_–TL_6_) that provide impedance matching between the antenna port, diode, and output load. These transmission lines are carefully optimized in Keysight Advanced Design System (ADS) to minimize the return loss (S_11_) and maximize power transfer at 1.8 GHz. Table 1 summarizes the optimized physical dimensions of these lines.

Figure 1 shows the layout of our proposed detector without band stop filter. In our proposed detector, we have used a low-cost SMS7630-079LF Schottky diode. For designing the detector, one capacitor (C1 = 24 pF: Murata Part No = CGQ1555C1H2R4BB01) is used. With these diode and capacitor, six TTLs (TL1–w1 = 1.79 mm, l1 = 7 mm; TL2–w2 = 1.89 mm, l2 = 20.68 mm; TL3–w3 = 2.10 mm, l3 = 2.06 mm; TL4–w4 = 1.79 mm, l4 = 1 mm; TL5–w5 = 2.81 mm, l5 = 21 mm TL6–w6 = 3.37 mm, l6 = 6.64 mm) have also been used for the design and implementation of this rectifier. The detector is realized on a Rogers RO4003C substrate with a dielectric constant (εᵣ) = 3.55, loss tangent (tan δ) = 0.0027, substrate height (h) = 0.817 mm, and copper thickness (t) = 17 µm. This substrate shows optimal performance for RF energy-harvesting use because it delivers better results than other materials for high-frequency testing while keeping manufacturing expenses under control. The via-ground connection which has a diameter of 0.8 mm and a width of 1 mm establishes a low-inductance return path that reduces parasitic effects while maintaining constant grounding. The detector has total physical dimensions of 58.61 × 11.45 mm, which makes it a compact and easy-to-integrate module. The input port is matched to a 50 Ω source impedance at the resonant frequency. The diode converts the incoming RF signal from the antenna into direct current, which the capacitor filters to generate stable output that supports low-power Internet of Things sensors and embedded edge devices. The testing reference design uses a single-band RF detector which operates from ambient energy-harvesting power to demonstrate its efficient low-cost operation and compact design and simple setup process.

### 3.2. Design of the Proposed Single-Band Detector with Band Stop Filter

**Figure 2:**
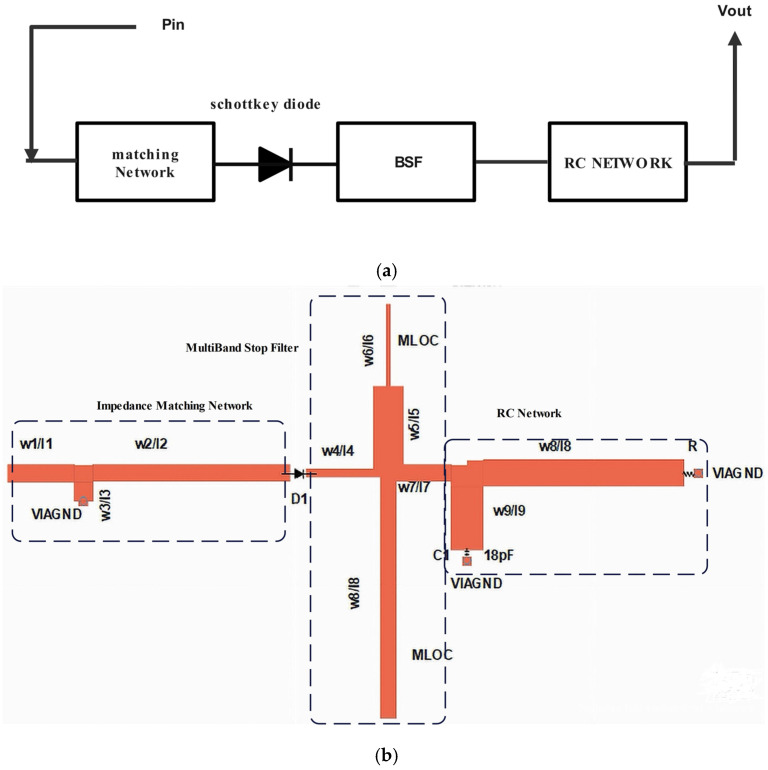
(**a**). Block diagram of proposed single-band rectifier with band stop filter designed at 1.8 GHz. (**b**). Layout of proposed single-band rectifier with band stop filter designed at 1.8 GHz (unit: mm).

The researchers integrated a band stop filter (BSF) into their rectifier system to enhance the basic detector performance. The rectification process creates harmonics which primarily include second- and third-order harmonics that reduce power conversion efficiency because they send energy back to the source and create impedance mismatches. By successfully reducing these undesirable elements, the rectifier’s linearity, sensitivity and conversion efficiency are enhanced. The BSF employed in the proposed design is based on a Stepped Impedance Resonator (SIR) structure, which provides compact geometry, precise stop band frequency control, and reduced insertion loss in the passband. The SIR-based BSF is embedded within the microstrip transmission line between the antenna feed and the rectifying diode to achieve selective rejection of harmonic frequencies while allowing efficient transmission at the fundamental frequency. The complete layout of the proposed rectifier with the integrated BSF is illustrated in Figure 2a.

Figure 2b shows the layout of our proposed broad single-band detector with band stop filter designed at 1.8 GHz detector. In our proposed detector, we used a low-cost SMS7630-79LF Schottky diode. For designing the rectifier, one capacitor (C1 = 18 pF: Murata Part No = GRM36C0G180JO50) is used. With this diode and capacitor, six TTLs (TL1–w1 = 1.8 mm, l1 = 7 mm; TL2–w2 = 1.79 mm, l2 = 20.64 mm; TL3–w3 = 1.92 mm, l3 = 2.06 mm; TL4–w4 = 0.8 mm, l4 = 7 mm; TL5–w5 = 3.16 mm, l5 = 8.18 mm TL6–w6 = 0.43 mm, l6 = 8.62 mm, TL7–w7 = 1.79 mm, l7 = 4.98 mm; TL8–w8 = 1.68 mm, l8 = 24.67 mm and TL9–w9 = 2.81 mm, l9 = 21 mm; TL10-w10 = 3.37 mm, l10 = 6.64 mm) have also been used for the design and implementation of this rectifier. Similar to the previous design, the SMS7630-079LF Schottky diode is used for rectification due to its high detection sensitivity at low input levels. The output smoothing is handled by a high-quality capacitor (C_1_ = 18 pF, Murata Part No. GRM36C0G180JO50) that filters high-frequency ripple components and delivers a steady DC output to the load. The enhanced topology consists of ten microstrip transmission lines (TL_1_–TL_10_) performing impedance matching, harmonic filtering, and power rectification functions. The detailed dimensions of these lines are provided in Table 2.

The detector design uses the same RO4003C substrate material as the reference design which maintains permanent dielectric and loss characteristics of εᵣ = 3.55 and tan δ = 0.0027 and height of 0.817 mm and thickness of 17 µm. The substrate height has been set to H = 0.817 mm and the copper thickness to T = 17 micron. The via-ground connection which has a diameter of 0.8 mm and a width of 1 mm provides grounding stability while reducing parasitic inductance. The circuit footprint achieved with BSF implementation maintains a size of 72.74 mm × 42.26 mm, which remains compact and simple to produce. The SIR-based BSF integration leads to two main benefits which are better harmonic suppression and improved impedance matching. The simulated results show that the BSF rectifier system delivers higher PCE and DC output voltage than the standard design. The system now shows better impedance stabilization because it re-radiates less harmonic power at its stable operating frequency of 1.8 GHz.

The single-band RF detector which we proposed has been optimized to work at 1.8 GHz for use in low-energy energy-harvesting applications, which include IoT nodes and wireless sensor networks. The researchers created two different systems which they studied through experiments:

- Configuration 1: Single-band detector without BSF, representing the baseline topology.
- Configuration 2: Enhanced detector with integrated SIR-based BSF, offering harmonic suppression and improved energy conversion.

Both designs employ the SMS7630-079LF Schottky diode due to its low threshold voltage and high sensitivity and are fabricated on Rogers RO4003C substrate, which ensures low dielectric loss and stable performance at microwave frequencies. The addition of the BSF greatly improves conversion efficiency, harmonic suppression and output stability, even when the baseline detector offers enough RF-to-DC conversion. The design’s usefulness for small and effective RF energy-harvesting modules used in autonomous IoT systems is highlighted by the enhancement that is made without adding to the circuit complexity or footprint.

### 3.3. Design of the Band Stop Filter (BSF) for Harmonic Suppression

The detector’s performance is usually degraded by the harmonic components that are created during the detection process. On the one hand, these harmonics diminish the sensitivity and accuracy of the detection and, on the other, they distort the input waveform, causing interference and power loss [5]. In order to alleviate these problems, a band stop filter (BSF) is placed in the detector circuit that suppresses the higher-order harmonics and, at the same time, allows the fundamental frequency to pass with a slight reduction in amplitude [32]. The detector’s signal purity is increased through this harmonic suppression and the system’s response is also improved. A Stepped Impedance Resonator (SIR) configuration is then chosen as the main component of the BSF in this paper. The SIR layout is especially helpful for microwave applications since it can produce a number of stopbands in a small space. It also enables the use of miniaturized RF detectors in portable and low-energy harvesting systems. The SIR not only allows for varying the location and width of the stop bands by changing the impedance and electrical length ratios of its transmission line sections but also provides the same facility of tuning parameters for conventional uniform impedance resonators. The ability to easily control the stop band location and widths makes it possible to realize compact filters with broad harmonic suppression capabilities.

### 3.4. Design and Theoretical Background Concept of the Band Stop Filter (BSF)

A Stepped Impedance Resonator (SIR) features a combination of high- and low-impedance transmission lines in its structure. If the correct impedance ratio is applied together with the proper lengths of the sections, the SIR will have multiple resonant frequencies with corresponding stop bands that very efficiently attenuate harmonic components. The main designing parameters for determining the location and intensity of resonant frequencies use the impedance ratio R = Z_1_/Z_2_ and electrical length ratio θ1/θ2 as their primary factors. The stopband frequency control uses these ratios to decrease their value which enables better harmonic rejection while maintaining the same structural size as before. The design requires the BSF to operate at its fundamental frequency f_0_ of 1.8 GHz, which matches the detector’s operational frequency. The SIR-based BSF designed in this way suppresses the frequencies 1.8 GHz, 3.6 GHz, 5.4 GHz and 7.2 GHz that are corresponding to the fundamental and its higher-order harmonics. The detector uses multiple stop bands to filter out unwanted harmonics while allowing only the fundamental frequency to pass through. This type of suppression produces a cleaner detected signal which leads to improved stability in DC output.

### 3.5. Structural and Substrate Design Parameters of the Band Stop Filter (BSF)

**Figure 3:**
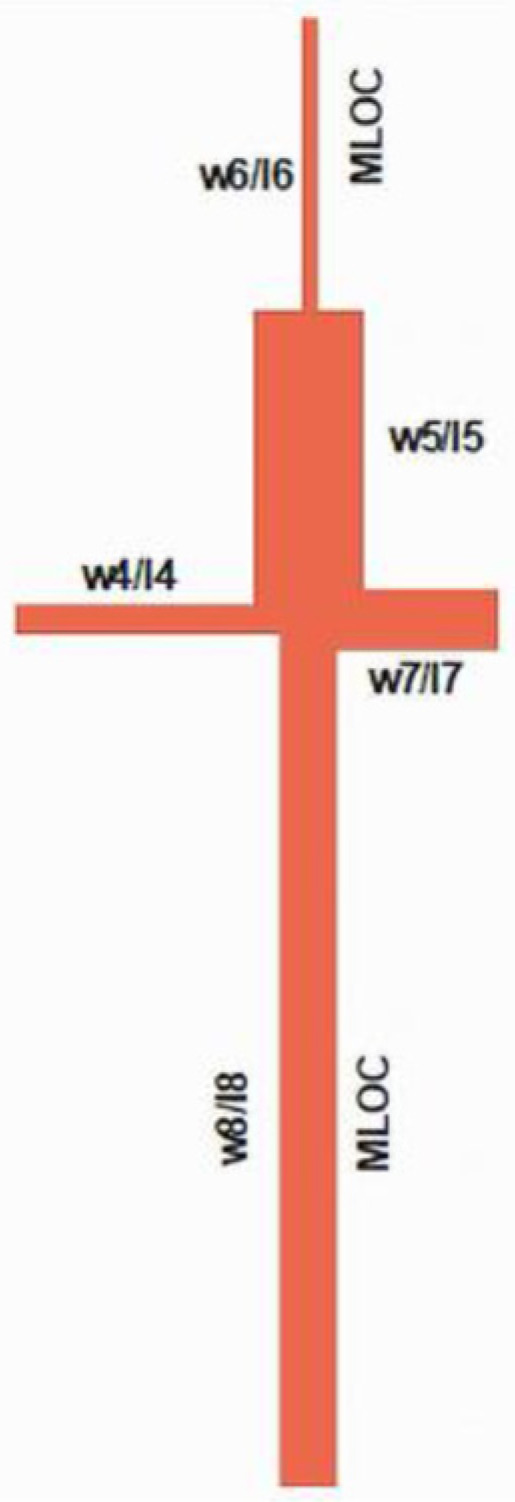
Microstrip line layout of BSF for designing the diode detector with a band stop filter to get higher sensitivity (unit: mm).

The BSF uses the Rogers RO4003C substrate which RF and microwave engineers prefer because it provides low dielectric loss and maintains mechanical stability while delivering exceptional performance at high frequencies. The substrate thickness measures 0.813 mm and its loss tangent (tan δ) value stands at 0.0027, while its relative permittivity (ε_r_) value equals 3.55. These features create high-Q factor resonances which allow stable frequency response and minimal signal loss to achieve effective harmonic suppression in detecting circuits. The proposed BSF uses four transmission line segments (TL4–TL7) which have been designed with specific geometric dimensions to achieve the desired resonant performance. The design includes an open-circuited stub which enhances rejection capabilities while achieving better impedance matching with the detector input. The stub functions as a quarter-wavelength resonator which operates at 90° for 1.8 GHz to enhance suppression of both fundamental and third harmonic signals. Moreover, its width and length are optimized to ensure smooth impedance transition between the BSF and detector, thereby minimizing reflection losses and maximizing power transfer. Figure 3 shows the layout of our proposed BSF for designing the diode detector, and the design parameters are summarized in Table 3.

For designing the band stop filter, we have used four TTLs (TL4–w4 = 0.8 mm, l4 = 7 mm; TL5–w5 = 3.16 mm, l5 = 8.18 mm; TL6–w6 = 0.43 mm, l6 = 8.62 mm; TL7–w7 = 1.79 mm, l7 = 3.98 mm, TL8–w8 = 1.68 mm, l8 = 24.67 mm) for the design and implementation of this rectifier. Each transmission line section contributes to specific resonant behavior, and their collective interaction creates multiple stop bands at desired frequencies. The combination of stepped impedances and the open stub results in a compact yet highly effective BSF design.

### 3.6. Design Optimization and Implementation of the Band Stop Filter (BSF)

The optimization of the BSF design was performed using a full-wave electromagnetic (EM) simulator to achieve accurate frequency response characteristics. Key parameters including the widths and lengths of the transmission lines, and the dimensions of the open stub, were iteratively refined to ensure that the stop bands aligned precisely with the harmonic frequencies. An open-circuited stub with 1.68 mm width and 24.67 mm length gives an electrical length of around 90° at 1.8 GHz, which is a part of the electrical circuit. The element itself elevates rejection thresholds at both fundamental and third-harmonic frequencies, which improves the spectral purity found at the detector output. The stub width is then arranged more to optimize impedance matching, thus ensuring that the RF energy coming from the source is at its maximum for conversion into DC or detected voltage signals. The use of stepped impedance sections leads to a great reduction in size, since the electrical length needed for a certain frequency can now be made shorter than that of uniform-line filters. This very fact makes the filter structure very appropriate for integration into small RF detectors of IoT nodes, wireless sensors, and portable communication devices.

### 3.7. Frequency and Simulation Response Analysis of the Band Stop Filter (BSF)

**Figure 4:**
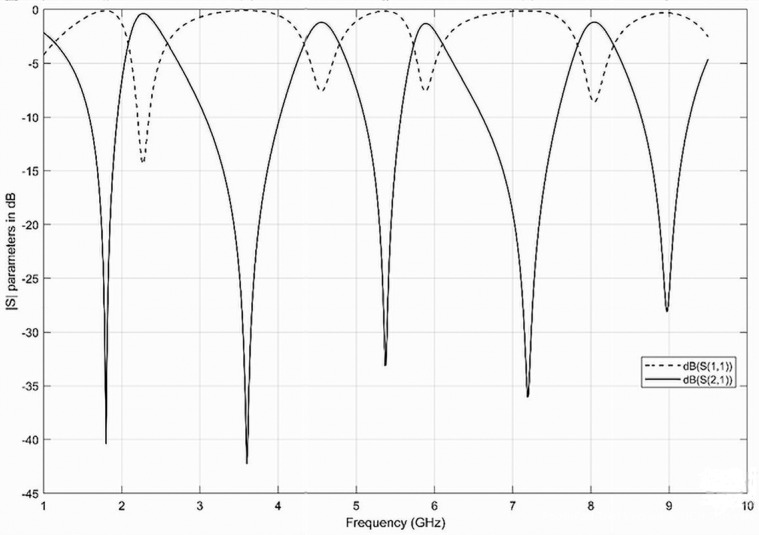
Simulated |S| parameters of the multi-band BSF.

The recommended BSF’s |S| parameters were established through full-wave 3D EM simulation. The S_21_ (transmission coefficient) response shows four distinct attenuation bands which represent both the fundamental wave and its higher frequency components that occur between 1.8 GHz and 7.2 GHz. The simulated S_11_ (return loss) demonstrates good impedance matching at the fundamental frequency because the BSF reflects only a small amount of energy within its designated operating range. The insertion loss inside each stop band surpasses 15 dB, as seen in Figure 4, indicating exceptional harmonic suppression capacity. The system maintains low insertion loss at non-resonant frequencies, which ensures that signal transmission will proceed without interruptions. The BSF effectively shields the detector from harmful harmonic interference while maintaining the strength of the original signal.

The band stop filter provides five stop bands which operate at 1.8 GHz, 3.6 GHz, 5.4 GHz and 7.2 GHz. A secondary open-stub which measures 1.68/24.67 mm serves to improve fundamental frequency rejection while allowing third-harmonic control through its 90° phase shift capability at 1.8 GHz. The output matching process needs open-stub width adjustment to achieve accurate performance results. Figure 4 displays the complete |S|-parameter results of the band stop filter, which were obtained through full-wave simulation. The system demonstrates over 15 dB rejection at its fundamental frequency and three subsequent harmonics. The simulation results reveal that the band stop filter maintains its frequency performance because of its design, which tolerates minor dimensional variations. The property is vital for practical production because manufacturers will encounter substrate thickness and etching precision changes during the manufacturing process.

### 3.8. Performance Integration and Impact with Detector of the Band Stop Filter (BSF)

The integration of BSF with the RF detector system results in better performance because it prevents unwanted harmonic signals from entering the sensitive detection area. The components produce power reflection with signal distortion because there is no harmonic suppression which results in diminished output voltage performance. The BSF system completely blocks all harmonic frequencies which enables it to produce a cleaner input signal, which in turn improves detection ability while keeping output voltage steady. The BSF system protects adjacent circuits and communication channels by reducing electromagnetic interference (EMI) through its capability to decrease harmonic radiation. The technology becomes essential for Internet of Things (IoT) and wireless sensor network environments because multiple devices function in close proximity to one another. The BSF maintains its budget-friendly design through its small planar design, which enables straightforward integration into detector systems that already exist without raising operational costs or requiring additional equipment. The BSF integration process improves system performance according to the simulation results and preliminary experiments. The proposed design for modern radio frequency detectors which operate at 1.8 GHz, and its related communication frequencies, achieves a balance between three design requirements of compact dimensions, effective harmonic suppression and impedance matching capabilities. The SIR-based BSF design system provides multiple stop bands through its compact design, which results in minimal insertion loss. The diode detector system achieves efficient harmonic component suppression through its combination with the detection system, which boosts overall detection accuracy. The recommended BSF serves as an ideal solution for upcoming Internet of Things detectors and wireless sensor nodes and low-power RF energy harvesting systems that require uninterrupted interference-free performance, because it demonstrates superior harmonic rejection and easy manufacturing and compatibility with planar technology [33].

## 4. Result

The performance evaluation of the proposed single-band RF detector working at 1.8 GHz along with and without the SIR-based band stop filter (BSF) has been done through extensive simulation and analysis. The results in terms of return loss, impedance matching sensitivity, output voltage and power conversion efficiency are presented. The whole simulation is done on Advanced Design System (ADS) software using practical diode models, substrate parameters and PCB layout effects.

### 4.1. Return Loss and Impedance Matching

**Figure 5:**
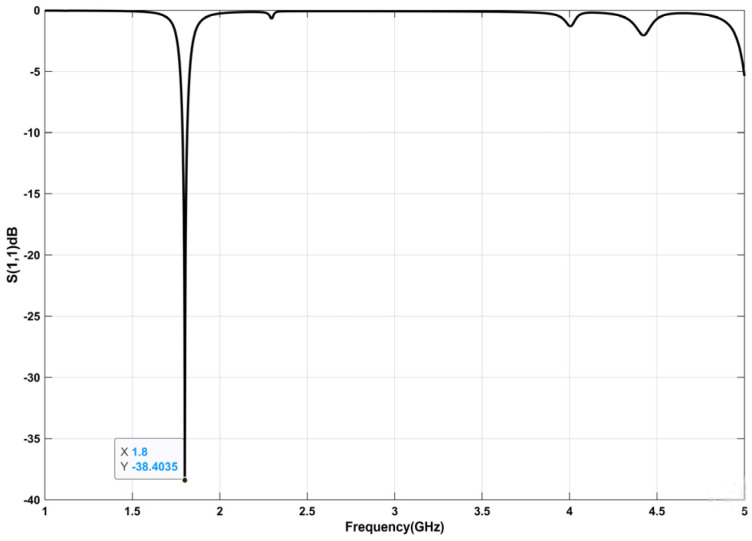
Variation in return loss (dB) with respect to frequency at fixed input power 0 dBm.

Impedance matching is a critical factor in determining the efficiency of RF energy harvesting systems. Poor matching leads to reflection losses and reduced power delivered to the rectifier. The simulated return loss (S11) for the proposed detectors at an input power of 0 dBm with different load resistances (1 kΩ, 1.5 kΩ, 9.53 kΩ) is presented in Figure 5 and summarized in Table 4.

**Figure 6:**
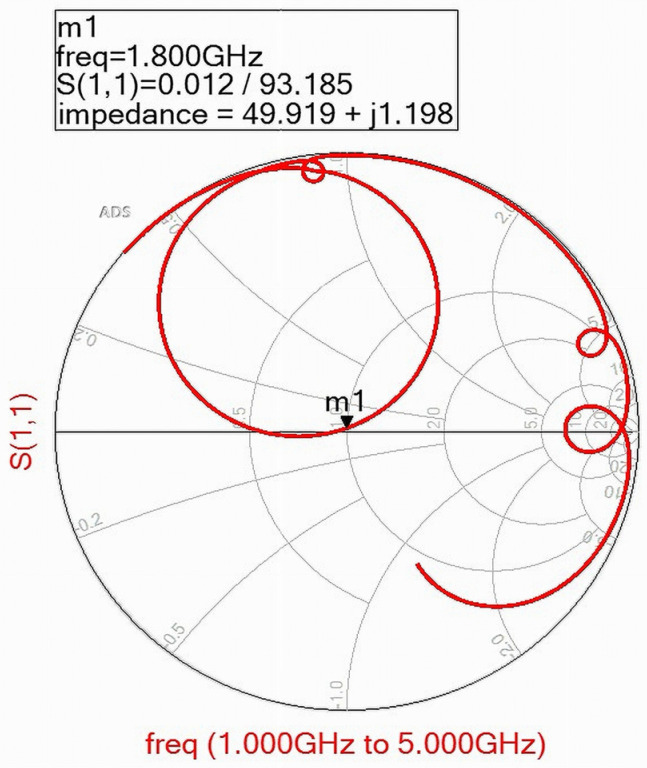
Representation of Smith chart for our proposed rectifier designed at 1.80 GHz frequency.

Figure 5 shows the variation in return loss (dB) with respect to frequency at fixed input power 0 dBm and load, which is also represented in Table 4. As can be seen in Figure 5 and Table 4, we have achieved −38.404 dB return loss at 1.8 GHz when considered with BSF. For the detector integrated with the SIR-based BSF, the return loss at 1.8 GHz consistently reaches approximately −38.4 dB, indicating excellent impedance matching. In comparison, the conventional detector without BSF achieves −44.16 dB. These values reflect near-ideal power transfer between the antenna and the rectifier, with minimal reflection. The Smith chart analysis (Figure 6) gives a clear view of the input impedance. The input impedance for the BSF-based detector at 1.8 GHz is 49.92 + j1.20 Ω, which is very much near to the standard 50 Ω reference, thus guaranteeing the maximum power transfer to the diode.

The Smith chart for our suggested rectifier, which is constructed at a frequency of 1.8 GHz, is shown in Figure 6. From Figure 6 we have noticed that at 1.8 GHz frequency, 49.92 + j*1.20 Ω impedance is obtained, which indicates the inductive impedance. Table 5 presents the input impedance values for different load conditions, confirming that the proposed design maintains excellent matching over a range of resistive loads, which is crucial for practical IoT and low-power sensor applications.

The improved impedance matching observed with BSF integration is attributed to the selective suppression of harmonic currents. By preventing the propagation of second and third harmonics, the BSF reduces reactive mismatches and stabilizes the input impedance, thereby enhancing energy transfer efficiency.

### 4.2. Sensitivity and Linearity

**Figure 7:**
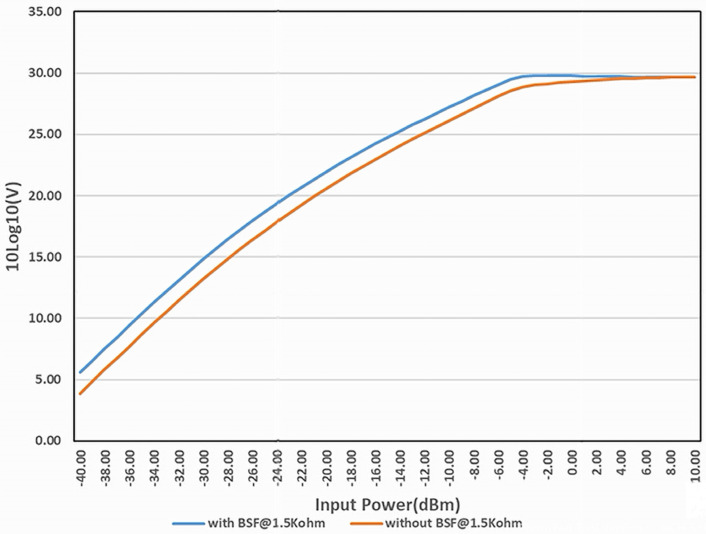
Results of square law region of diode detector with BSF and conventional detector.

The sensitivity and linearity of an RF detector determine its ability to accurately convert low-level RF signals into measurable DC voltage. Figure 7 illustrates the square law region characteristics of the proposed detectors.

Figure 7 highlights the square law operating range of the proposed BSF-based diode detector, which is evident through its comparison with the standard detector. The proposed device shows an extended square law region which operates in a more linear fashion while delivering better output sensitivity at low input power. The band stop filter operating system enables the diode detector to achieve improved square law performance because it successfully stops radio frequency interference and blocks higher-frequency harmonic signals. The system achieves better DC component extraction which results in higher voltage sensitivity and a broader square law operating range. The proposed device operates effectively because it has better low power detection capabilities and a wider square law operating range and it minimizes radio frequency interference.

The band stop filtering process leading blocks harmonic signal results with better rectification efficiency, which leads to this enhancement. The proposed device detects signals with greater efficiency while maintaining better signal integrity and increasing energy transformation capabilities when it operates with minimal power. The device offers these benefits, which make it suitable for detecting low-power radio frequency signals and collecting energy from ambient sources.

From the above figure, it is observed that the detector with integrated BSF increases the linearity as compared to conventional diode detectors. The above graph shows the sensitivity results which indicate that sensitivity without BSF operates at 1.8 mV/dBm, while the introduction of BSF increases sensitivity to 2.2 mV/dBm.

In the low input power region (−35 dBm to −25 dBm), the output voltage of the BSF-integrated detector exhibits a nearly linear relationship with input power, confirming operation within the square law regime. The output sensitivity of the detector (without BSF) is 1.8 mV/dBm, whereas incorporating the SIR-based BSF integration increases it to 2.2 mV/dBm. The detector maintains a predictable and linear response despite low RF input levels by decreasing the interference from higher-order harmonics. This is crucial for Internet of Things sensors, which often operate under weak ambient RF signals. Furthermore, the BSF-enhanced linearity ensures minimal distortion in the output DC signal, enabling reliable powering of sensitive electronic loads without the need for additional voltage regulation circuits.

### 4.3. Output Voltage Characteristics

**Figure 8:**
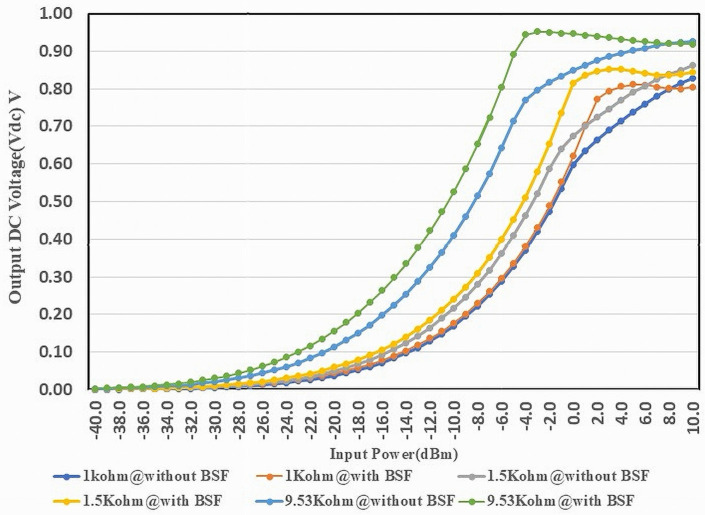
Variation in output DC voltage of diode detector with BSF with respect to input power (dBm) at frequency 1.80 GHz and also for varying load.

Figure 8 shows the variation in output DC voltage with respect to input power (dBm) at frequency 1.80 GHz and also for varying load, which is also represented in Table 6. At 1.80 GHz frequency, we have achieved maximum 0.95 V DC output voltage for the input power 0 dBm and 9.53 kΩ load.

**Figure 9:**
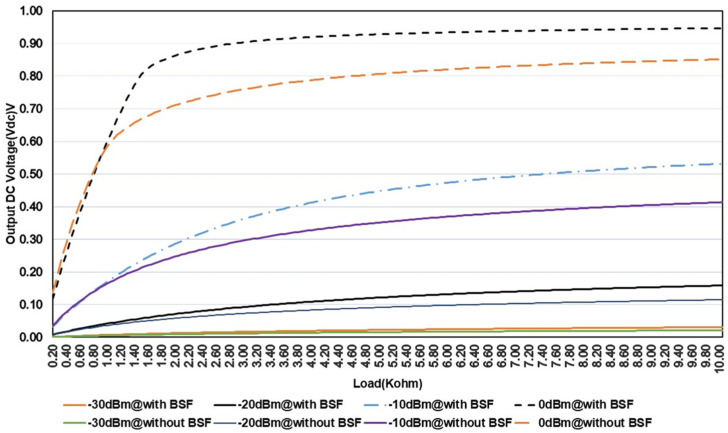
Variation in output voltage with respect to load at fixed frequency 1.8 GHz and for varying input power.

The above figure, Figure 9, shows the variation in output voltage with respect to load at a fixed frequency of 1.8 GHz and for varying input power. From Figure 9, it can be noticed that the maximum output voltage 0.95 V is obtained with input power 0 dBm and load 9.53 kΩ, respectively.

Both detectors exhibit a monotonic increase in output voltage with increasing RF input power, consistent with diode rectification theory. For the detector with BSF, a maximum DC voltage of 0.95 V is achieved at an input power of 0 dBm with a load of 9.53 kΩ. Across all tested load conditions, the BSF-based design consistently produces higher output voltages than the conventional detector. The improved impedance matching and decreased harmonic losses brought forth by BSF integration are responsible for this improvement. The results confirm that the proposed design is capable of supplying sufficient DC voltage to power low-power IoT sensor nodes and embedded devices directly from ambient RF energy, eliminating or reducing dependence on conventional batteries.

### 4.4. Power Conversion Efficiency (%)

**Figure 10:**
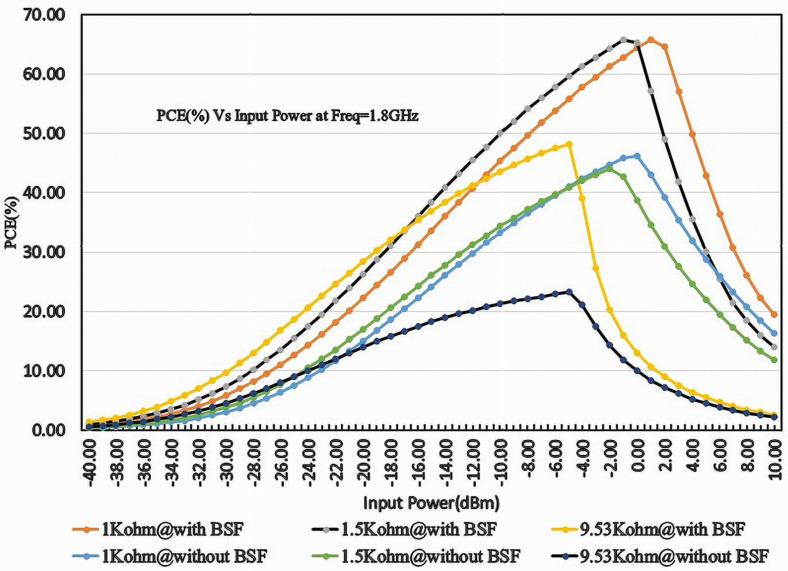
Variation in efficiency of diode detector with BSF with respect to input power (dBm) at fixed frequency of 1.8 GHz and for varying load.

**Figure 11:**
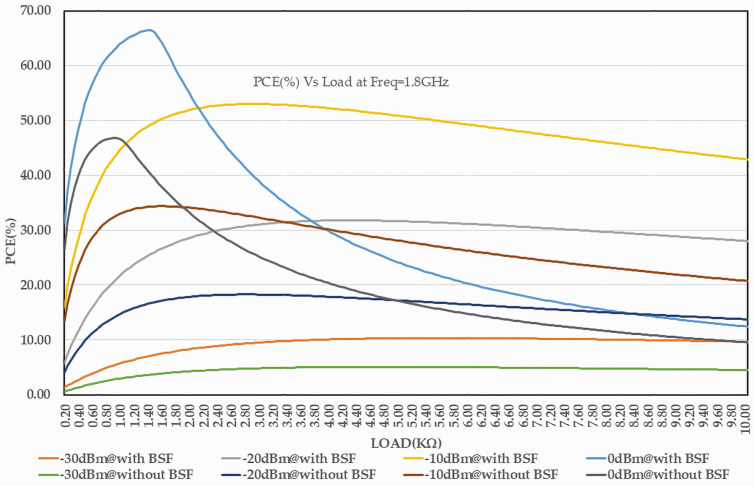
Variation in efficiency with respect to load at fixed frequency 1.8 GHz and for varying input power and varying load.

The power conversion efficiency (PCE) is a critical metric for RF energy-harvesting circuits, defined as the ratio of output DC power to input RF power. The efficiency of an RF detector depends mainly on three factors, which include the properties of its rectifying element, its impedance matching capabilities, its load optimization methods and its harmonic suppression techniques. The performance of low-power RF detection systems depends mainly on three parameters, which include diode threshold voltage, junction capacitance and square law operation, while proper matching and filtering ensure maximum RF power transfer to the rectifier. The proposed device improves power conversion efficiency by ensuring efficient impedance matching between the RF source and the nonlinear rectifying diode, thereby minimizing reflection losses and maximizing RF power delivery to the rectifier. The optimized circuit topology improves the rectification process because it operates better with low input power, which ambient RF energy-harvesting applications need. The system achieves higher efficiency because it converts a greater portion of incoming RF energy into usable DC electrical power. The simulated PCE behavior for the proposed detectors is presented in Figure 10 and Figure 11 and detailed in Table 7.

Figure 10 shows the variation in efficiency with respect to input power (dBm) at a frequency of 1.8 GHz and also for varying load, which is represented in Table 7.

As can be seen in Figure 10 and Table 7, we achieved our highest efficiency of 65.28%, which occurs at 0 dBm and 1.5 kΩ load, designed for 1.8 GHz operation. The efficiency measurements show maximum efficiency at 9.53 kΩ load for power levels between −30 dBm and −20 dBm. The proposed design operates at 1.8 GHz and achieves power conversion efficiency (PCE) above 30% over a wide input power range from –20 dBm to +5 dBm and maximum peak PCE = 65.28% and input power = 0 dBm.

The design works at 1.8 GHz and achieves a power conversion efficiency (PCE) of 30% or higher across a power input range from −20 dBm to +5 dBm while reaching its highest PCE value of 65.28% at 0 dBm input power. The rectifier has been designed to function correctly under low input power conditions because it achieves high sensitivity with a performance measurement of 2.2 mV/dBm at a −35 dBm input. The 30% power conversion efficiency (PCE) serves as a practical minimum requirement for RF rectifiers and energy-harvesting circuits that operate at low input power levels. The RF energy-harvesting systems require this level of efficiency because their power capacity is extremely limited and efficiency rates below this point will not produce enough DC output for practical applications or power-management systems. The design achieves stable performance under varying input power conditions while maintaining efficiency levels above 30% between −20 dBm and +5 dBm and reaching its highest efficiency point of 65.28% at 0 dBm. The proposed rectifier combines low power optimization with wide dynamic range operation which allows it to function in both ambient RF energy-harvesting environments and low-power RF detection scenarios.

Figure 11 shows the variation in efficiency with respect to load at a fixed frequency of 1.8 GHz and for varying input power. A maximum 65.28% efficiency is achieved at 1.5 kΩ load and 0 dBm input power. Figure 11 shows how power conversion efficiency (PCE) changes with different load resistance R_L_ values at 1.8 GHz and various input power levels. The figure demonstrates its practical value by showing the load resistance range that delivers maximum PCE performance under real-world operational conditions. The system maintains consistent performance throughout different input power levels, which proves necessary for ambient RF energy-harvesting applications.

The proposed detector operates with a 1.5 kΩ load, which serves as its testing and performance assessment base. The 9.53 kΩ resistance was employed only during preliminary parametric simulations to examine the effect of load variation and was not intended as the operating load. The load resistance value of 1.5 kΩ was selected for the detector because it represents a more practical and stable operating condition for RF detection and low-power measurement, which exceeds the performance of 9.53 kΩ. The selection of 1.5 kΩ load resistance enables detector operation with faster response times and better operational stability, while maintaining actual detection performance at this value. The selected load resistance establishes a balance point which determines three operational characteristics that depend on the specific application needs of the system. The study used a lower load resistance to achieve both faster response times and practical low input power level detection while still maintaining acceptable sensitivity levels.

The proposed detector with the integrated BSF achieves a peak PCE of 65.28% at 0 dBm input power and a load of 1.5 kΩ, surpassing the conventional single-diode design, which achieves 60.71% under identical conditions. The efficiency improvement arises from the following:

1. Harmonic suppression reducing re-radiated power and parasitic losses.
2. Improved impedance matching maximizing power transfer from the antenna to the rectifier.
3. Minimized reflection and resistive losses due to optimized microstrip line dimensions and proper VIA grounding.

The PCE decreases at higher load resistances due to reduced current flow in the diode and the non-linear response of the rectifying element. Nevertheless, as compared to the traditional architecture, the BSF-based detector continues to perform better even at greater resistances.

### 4.5. Comparative Analysis

With bigger or more intricate multi-stage architectures, the majority of current devices running close to 1.8 GHz have PCE values below 55%. The suggested detector on the other hand uses a compact single-series diode topology to achieve 65.28% PCE highlighting its high performance, affordability and ease of use. Furthermore, the suggested design incorporates harmonic suppression within the same planar footprint (53.68 × 17.39 mm^2^) without the need for multi-layer structures or extra passive components, which is beneficial for embedded energy-harvesting and Internet of Things applications where manufacturability, cost and size are crucial limitations. The findings unequivocally show that adding an SIR-based BSF to a single-band RF detector resulted in observable gains in return loss, sensitivity, output voltage and overall energy harvesting efficiency. These improvements make the suggested detector a viable and effective option for autonomous embedded devices, IoT nodes and low-power wireless sensor networks. As can be seen in Table 8.

## 5. Conclusions

The research utilizes a single-band passive RF detector which operates at 1.8 GHz through its dual-diode Schottky design and compact impedance-matching system. The proposed rectifier achieves a peak RF-to-DC conversion efficiency of 65.28% at 0 dBm, while maintaining an efficiency above 30% across an input power range that extends from −20 dBm to +5 dBm. The detector operates under weak electromagnetic interference conditions because it achieves a low-power voltage sensitivity of 2.2 mV/dBm at −35 dBm without needing RF or baseband amplifiers. The integrated band stop filter (BSF) functions as an essential component which helps to suppress higher-order harmonics and block RF leakage from the output while enhancing impedance matching at low input power levels. The square law region output voltage slope increases, which results in greater sensitivity and higher conversion efficiency. The proposed rectifier design enables effective power detection through ambient RF energy harvesting and low-power RF detection.

## 6. Future Scope

The present design contains multiple ways to enhance its performance, through upcoming improvements which will lead to better results in advanced communication and sensing systems. The detector will undergo testing in upcoming studies to explore its ability to operate across dual or multiple frequency bands through advanced research methods. Adaptive impedance matching networks together with reconfigurable filtering structures will enhance conversion efficiency because these systems can modify their output based on different input requirements. The proposed circuit requires practical implementation, which includes its manufacturing and measurement testing, because this process will validate simulated results and demonstrate product performance in real-world testing. The detector enables hybrid energy-harvesting platforms for IoT networks which operate without batteries when combined with antenna arrays or frequency-selective surfaces. Future research will develop new semiconductor materials with advanced substrates, which will lower losses while improving thermal stability, because these factors are crucial for embedded systems and biomedical systems that require long-term operation.

The current research investigates microwave frequency design and analysis methods but demonstrates that its proposed device design can be used with millimeter wave systems through proper selection of high-frequency components and substrates and fabrication methods. The system achieves superior performance through its implementation of millimeter wave Schottky diodes, low-loss substrates and accurate lithographic manufacturing methods. The proper measurement methods for millimeter wave band experiments include on-wafer and wave guide measurement techniques.

## Figures and Tables

**Table 1:** Parameter of proposed single-band detector without band stop filter.

Microstrip Line	W (mm)	L (mm)
TL1	w1 = 1.79	l1 = 7
TL2	w2 = 1.89	l2 = 20.68
TL3	w3 = 2.10	l3 = 2.06
TL4	w4 = 1.79	l4 = 1
TL5	w5 = 2.81	l5 = 21
TL6	w6 = 3.37	l6 = 6.64

**Table 2:** Parameter of proposed detector with integrated band stop filter.

Microstrip Line	W (mm)	L (mm)
TL1	w1 = 1.8	l1= 7
TL2	w2 = 1.79	l2 = 20.64
TL3	w3 = 1.92	l3 = 2.06
TL4	w4 = 0.8	l4 = 7
TL5	w5 = 3.16	l5 = 8.18
TL6	w6 = 0.43	l6 = 8.62
TL7	w7 = 1.79	l7 = 4.98
TL8	w8 = 1.68	l8 = 24.67
TL9	w9 = 2.81	l9 = 21
TL10	w10 = 3.37	l10 = 6.64

**Table 3:** Parameters of the BSF for designing the diode detector.

Microstrip Line	W (mm)	L (mm)
TL4	w4 = 0.8	l4 = 7
TL5	w5 = 3.16	l5 = 8.18
TL6	w6 = 0.43	l6 = 8.62
TL7	w7 = 1.79	l7 = 3.98
TL8	w8 = 1.68	l8 = 24.67

**Table 4:** Variation in return loss (dB) with respect to frequency at fixed input power 0 dBm.

	1 kΩ	1.5 kΩ	9.53 kΩ
Freq (GHz)	s11 (dB)	s11 (dB)	s11 (dB)
1.8 (without BSF)	−44.16	−40.310	−36.038
1.8 (with BSF)	−38.403	−38.404	−38.404

**Table 5:** Representation of Smith chart for our proposed single band detector designed at 1.8 GHz.

Load (Ω)	1 kΩ	1.5 kΩ	9.53 kΩ
**Input Power (dBm)**	**0**	**0**	**0**
**Freq (GHz)**	**Zin**	**Zin**	**Zin**
1.8 (without BSF)	50.44 − j0.44	50.53 − j0.81	50.62 − j1.46
1.8 (with BSF)	49.92 + j1.20	49.92 − j1.20	49.92 + j1.20

**Table 6:** Parameters of output DC voltage for the variation in input power (dBm) at frequency 1.80 GHz and also for varying load.

Freq (GHz)	1.8	1.8	1.8	1.8	1.8	1.8
**Load (Ω)**	**1 k**	**1.5 k**	**9.53 k**	**1 k**	**1.5 k**	**9.53 k**
**Input Power (dBm)**	**Vdc (Volt) Without BSF**			**Vdc (Volt) with BSF**		
−30	0.01	0.01	0.03	0.01	0.01	0.03
−20	0.04	0.05	0.15	0.04	0.06	0.16
−10	0.17	0.22	0.51	0.18	0.24	0.53
0	0.60	0.67	0.96	0.62	0.82	0.95

**Table 7:** Parameters of efficiency with respect to input power (dBm) at fixed frequency of 1.8 GHz and also for varying load.

Freq (GHz)	1.8	1.8	1.8	1.8	1.8	1.8
**Load (Ω)**	**1 k**	**1.5 k**	**9.53 k**	**1 k**	**1.5 k**	**9.53 k**
**Input Power (dBm)**	**PCE (%) Without BSF**			**PCE (%) with BSF**		
−30	3.06	3.83	4.57	5.89	7.38	9.77
−20	15.01	17.02	14.02	22.25	26.36	28.40
−10	33.25	34.36	21.27	45.29	49.94	43.57
0	46.27	38.66	9.95	64.38	65.28	13.00

**Table 8:** Comparison of results of our proposed work with respect to other works.

Ref	Freq	Substrate	Topology	Diode	Load	Efficiency
16	2.45 GHz	FR4	voltage doubler	HSMS2852	10 kΩ	52%@0 dBm
17	2450 MHz	NR	Single diode	SMS7630-0LF		55.7%@8 dBm
19	5.8 GHz	Rogers 4350B	Single-stage rectifier	HSMS 286C	0.6 kΩ	59.6% @16.7 dBm
21	2.4 GHz	N.R	Series RF rectifier	SMS7630	NA	25.33@−20 dBm
22	5.8 GHz	N.R	Voltage doubler	SMS7630	1 kΩ	51.8%@ (−20 to 15) dBm
23	2.4 GHz	NR	Single- and multi-stages Voltage-doubler Rectifier	ASPAT tunnel diode	10 kΩ	15%@−30 dBm
24	0.9 GHz	NR	Class-E/F2 shunt rectifier	HSMS2850	4.3 kΩ	50%@0 dBm
This work	1.8 GHz	FR4	Single-series diode	SMS7630-0LF	1.5 kΩ	Max 65.28%@0 dBm

N.R—Not Reported.

## Data Availability

The original contributions presented in this study are included in the article. Further inquiries can be directed to the corresponding author.
